# Mortality trend alteration of thoracic injury after rapid response trauma team establishment

**DOI:** 10.1186/cc11061

**Published:** 2012-03-20

**Authors:** K Chittawatanarat, C Ditsatham, K Chandacham, T Jirapongchareonlap, N Chotirosniramit

**Affiliations:** 1 Chiang Mai University, Chiang Mai, Thailand

## Introduction

The Department of Surgery, Faculty of Medicine, Chiang Mai University established a rapid response trauma team (RRTT) in July 2006. The aims of this study were to verify mortality rate alteration after setting up the RRTT.

## Methods

We retrospectively collected data between January 2004 and September 2009. The month before July 2006 was defined as before RRTT and after July 2006 as after RRTT. The monthly mortality rate, severity injury score (ISS) and demographic data were collected.

## Results

A total 951 patients were included (427 (30 months) before RRTT and 524 (39 months) after RRTT). Of these, 83 patients (8.8%) were dead after admission and analyzed for characters of mortality. The average age of mortality patients was 38.7 ± 16.3 years. Male was the predominant gender. The most common mechanism of injury was a motorcycle accident. Although there were no differences of character and mechanism of injuries between the two periods, patients associated with maxillofacial injury had significant lower mortality after RRTT (28.5% vs. 10.5%; *P *= 0.04). However, the after RRTT group had significantly higher occurrence of urinary complication and acute renal failure. The average adjusted monthly mortality rate was lower after RRTT (9.0 ± 6.1 vs. 6.9 ± 4.0%). Time series analysis between two periods demonstrated a decrease trend in monthly mortality after RRTT (coefficient (95% CI) = -0.61 (-1.13 to -0.23); *P *< 0.01)).

## Conclusion

Rapid response trauma team establishment could decrease the mortality trend. A protective effect was predominant in patients associated with maxillofacial injury.

